# Supplementary material for: The interactome of intact mitochondria by cross-linking mass spectrometry provides evidence for coexisting respiratory supercomplexes
Source: Mol Cell Proteomics. 2017 Dec 8;17(2):216–32. doi: 10.1074/mcp.RA117.000470 (PMC5795388; doi:10.1074/mcp.RA117.000470)
Supplement: Supplemental Data [file supp_RA117.000470_133922_0_supp_23978_fzffwf.zip › spectra_annotation/mito_spectra_annotation/58-1-13-1-7-1.pdf]

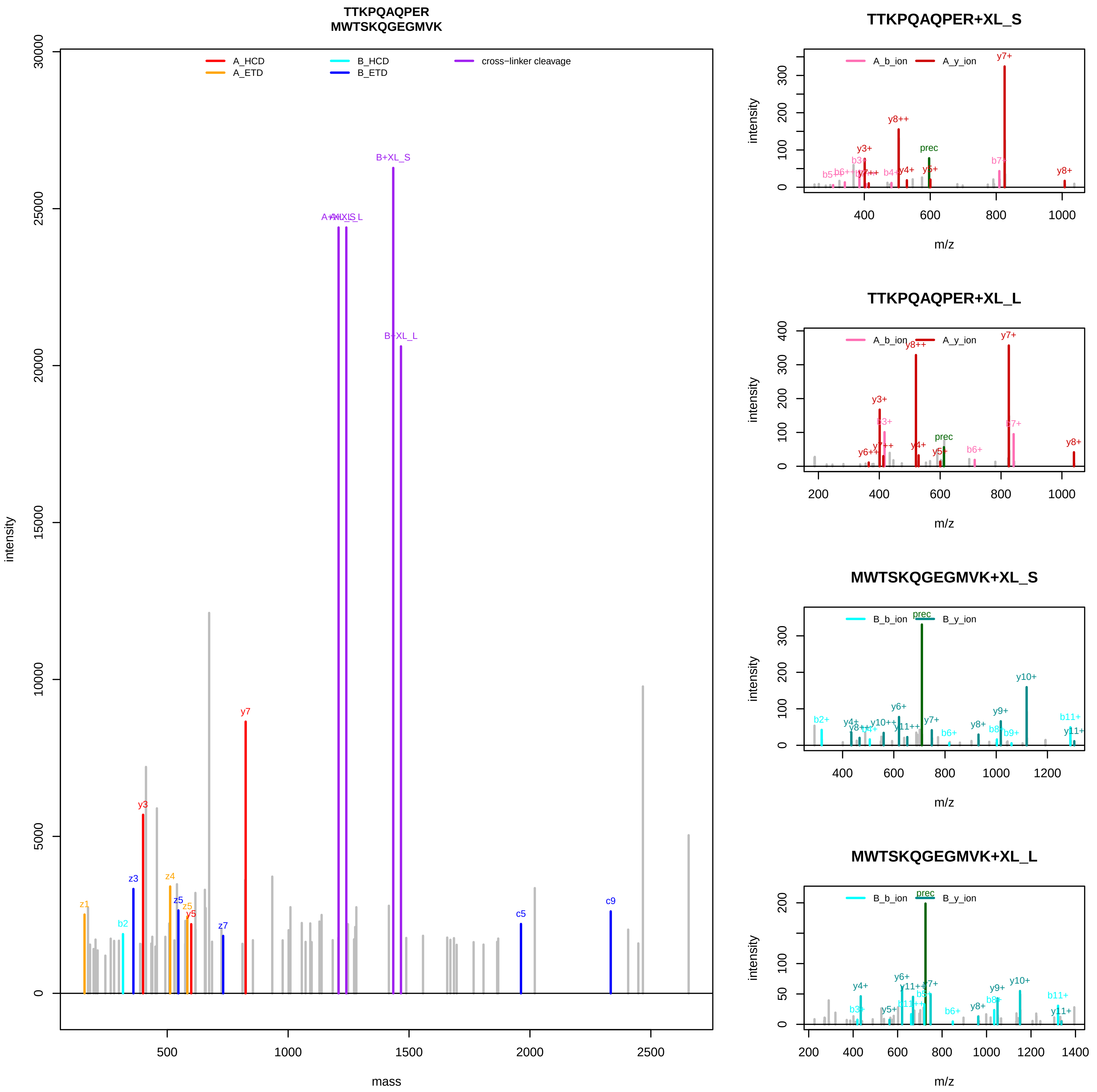
TTKPQAQPER
MWTSKQGEGMVK
A_HCD
A_ETD
B_HCD
B_ETD
cross−linker cleavage
B+XL_S
B+XL_L
y7
y3
z1
b2
z3
z4
z5
y5
z7
c5
c9
intensity
mass
TTKPQAQPER+XL_S
A_b_ion
A_y_ion
y7+
y8++
y3+
prec
b7+
y8+
m/z
TTKPQAQPER+XL_L
MWTSKQGEGMVK+XL_S
B_b_ion
B_y_ion
y10+
y6+
y9+
b11+
y11+
MWTSKQGEGMVK+XL_L
